# Quantitative sensory testing in dogs with spontaneous osteoarthritis

**DOI:** 10.3389/fpain.2025.1518725

**Published:** 2025-04-14

**Authors:** James Russell Hunt, David Knazovicky, Megan Goff, John Harris, Toby G. Knowles, Masataka Enomoto, Michael Mendl, Becky Whay, B. Duncan X. Lascelles, Joanna C. Murrell

**Affiliations:** ^1^ Bristol Veterinary School, University of Bristol, Bristol, United Kingdom; ^2^ Department of Clinical Sciences, College of Veterinary Medicine, North Carolina State University, Raleigh, NC, United States; ^3^ Arthritis Research UK Pain Centre and Division of Animal Sciences, School of Biosciences, University of Nottingham, Loughborough, United Kingdom; ^4^ Translational Research in Pain, College of Veterinary Medicine, North Carolina State University, Raleigh, NC, United States; ^5^ Natural Sciences, University of Galway, Galway, Ireland; ^6^ Center for Comparative Pain Research and Education, North Carolina State University, Raleigh, NC, United States; ^7^ Center for Pain Research and Innovation, UNC School of Dentistry, Chapel Hill, NC, United States; ^8^ Department of Anesthesiology, Center for Translational Pain Research Medicine, Duke University, Durham, NC, United States; ^9^ Bristol Vet Specialists, Bristol, United Kingdom

**Keywords:** dog, spontaneous osteoarthritis, quantitative sensorial testing (QST), central sensitisation, pain

## Abstract

**Objective:**

To investigate changes in somatosensory sensitivity in dogs with spontaneous osteoarthritis (OA) and pain of the stifle or hip, compared to a group of healthy control dogs.

**Study design:**

A non-randomised, non-blinded, prospective research study.

**Animals:**

30 control, 51 OA-pain, and 31 OA-pain dogs receiving NSAIDs

**Methods:**

A range of noxious and non-noxious quantitative sensory testing (QST) modalities were applied. Dogs were tested twice, one month apart. Two sites were tested at each visit: a distal site located on the cranial aspect of the mid metatarsus and a primary site, lateral to the patella (in dogs with stifle OA) or craniodorsally to the greater trochanter (in dogs with coxofemoral OA). Control dogs were tested at appropriate primary sites to produce the same proportion of animals being tested at stifle or hip as those in the OA group. The order in which non-nociceptive and nociceptive tests were performed was randomized for each test site for each animal, although nociceptive tests were always performed after non-nociceptive tests. Feasibility for performing the tests was assessed for the final 45 dogs recruited to the study. The hierarchical structure of the QST testing data was accounted for within the statistical analysis by employing general linear modelling within a multilevel modelling framework using the MLwiN statistics package.

**Results:**

Osteoarthritis category was not a major determinant of QST outcome measures for the majority of modalities evaluated. In the few modalities in which OA category was determined to be a significant predictor variable, the results were not consistent with previously reported data. The novel, non-nociceptive tests employed overall suggested non-noxious hypoesthesia in association with OA pain. The feasibility of performing QST assessments was relatively low compared to previous studies.

**Conclusions:**

and clinical relevance: In a clinical environment, the variability in feasibility of performing QST between dogs may be sufficient to confound changes in QST outcome measures associated with spontaneous OA.

## 1 Introduction

Quantitative sensory testing (QST) has been widely employed in the investigation of somatosensory alterations associated with central sensitisation in man (1–3) and evaluated as a means of predicting analgesic response (4). Sensory profiling, permitting sub-grouping of patients, has been demonstrated to identify treatment responders in some pain conditions (5). Quantitative sensory testing in the form of nociceptive response testing is well established as a fundamental outcome measure in pre-clinical analgesic and pain research in non-human species (6–10), and recently QST has begun to be investigated in research units for utility in the assessment of companion animals (11–13), both as a potential veterinary clinical tool and as a pre-clinical measure in spontaneous disease companion animal models. Knazovicky et al. (2016) (14) reported widespread somatosensory gain of function in association with OA in dogs providing evidence of central sensitisation in this population of dogs and strengthening the validity of canine spontaneous OA as a model for human OA. Significant clinically problematic aspects of central sensitisation in man relate to inappropriate perception of normally non-noxious stimuli as painful (15, 16) (allodynia), an aspect of QST which has previously not been explored in dogs suffering from spontaneous OA. Loss of sensory function, identified by increased (e.g., vibration or mechanical) detection threshold, has also been reported in association with central sensitisation in man (15); the assessment of response to the application of non-noxious stimuli to animals may be able to detect such sensory loss.

Environmental factors affecting QST data collected from dogs are not well described. Investigators have reported using a dedicated or quiet space with a relatively constant temperature (17, 18) in order to minimise distraction for the dogs undergoing testing. However, for widespread adoption of the technique as a pre-clinical and clinical tool, QST measurement should be feasible and demonstrate repeatable results in common clinical veterinary settings.

Our aim was to evaluate the utility of a wide range of QST measures in client-owned dogs suffering from spontaneous OA within an environment comparable to a veterinary practice and compare these recordings with data from a matched control group. Using a veterinary practice setting to collect data would be conducive to the ultimate aim of recruiting large numbers of dogs into studies. The studies reported here were part of a larger project (19) aimed at understanding the association between neurophysiological measures of central sensitisation and QST measures. We hypothesised that dogs suffering from OA would demonstrate altered QST measurements compared with normal dogs, showing hyperalgesia and allodynia to noxious and non-noxious stimuli respectively.

## 2 Material and methods

### 2.1 Ethics

The study was conducted under the terms of the Animal (Scientific Procedures) Act (A(SP)A, 1986, as amended 2013, licence number PPL 30/3157, and the experimental protocol was approved by the University of Bristol Animal Welfare and Ethical Review Body.

### 2.2 Animals

Owners of eligible dogs were asked to attend a screening appointment, at which the purpose and procedures of the study were explained verbally and in writing, and signed consent to participate was obtained prior to any study procedures being performed. Microchip details were confirmed as a means of permanently identifying participating dogs to comply with the terms of the A(SP)A.

Dogs underwent physical and musculoskeletal examinations by a veterinarian (JRH) as detailed previously (19), with scores being assigned for degree of lameness, mobility impairment, OA burden, and joint pain burden. Dogs exhibiting pain on joint manipulation of the stifle or hip uni- or bi-laterally were recruited to the osteoarthritis pain group (OA-pain). During recruitment, a large number of dogs attending for screening were already receiving treatment with non-steroidal anti-inflammatory drugs (NSAIDs) for OA-pain and the decision was made to recruit these cases to a second group, OANSAID. Control dogs of similar age and body weight to previously reported OA populations (20) were recruited.

### 2.3 Study protocol

Owners were asked to complete the ACVS (American College of Veterinary Surgeons) Canine Orthopaedic Index (21), the Helsinki Chronic Pain Index (HCPI) (22), the Liverpool Osteoarthritis in Dogs questionnaire (LOAD) (23), the Canine Brief Pain Inventory (CBPI) (24), and the Sleep and Night time Restlessness Evaluation (SNORE) (18). Jugular blood samples were obtained and submitted for routine biochemistry and haematology. As part of the overarching project, dogs underwent anaesthesia, radiography, and nociceptive withdrawal testing as previously described (19). One week following anaesthesia dogs revisited the study center to complete the first QST protocol, which was repeated a second time 4 weeks later.

### 2.4 Quantitative sensory testing protocol

Quantitative sensory testing was performed in one of two rooms. The rooms resembled consulting rooms used in veterinary practice for clinical work. One room measured 5.1 × 4.2 m and housed windows along one side and one door, the second room measured 9.2 × 8 m and natural illumination was provided by roof windows. Owners of dogs were not present during QST testing.

#### 2.4.1 Environment

**Figure 1:**
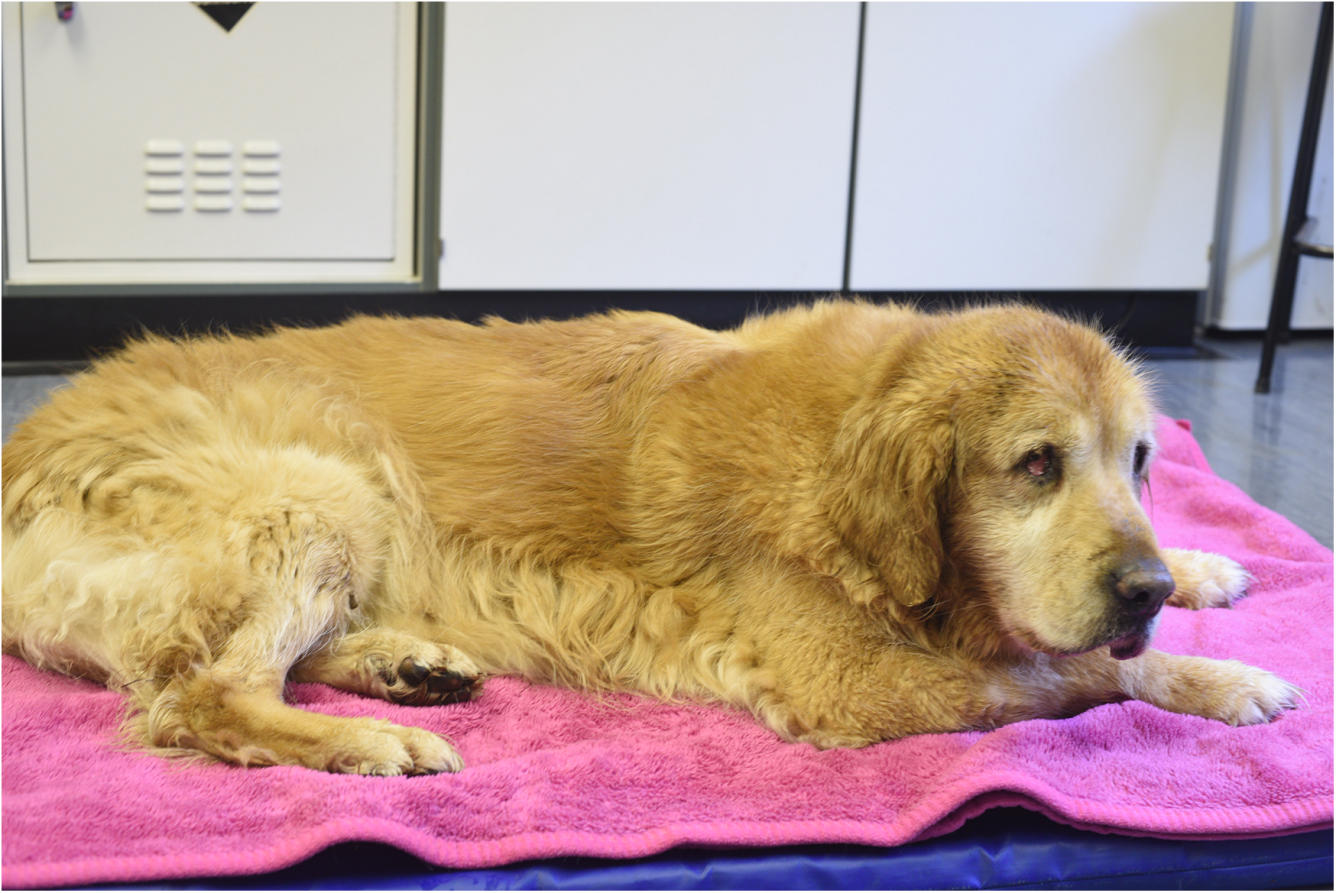
Dog lying on a mat in preparation for QST assessments.

During testing, rooms were maintained at between 20 and 25°C. Dogs spent 15 min at the beginning of each session acclimatising to the testing room with the investigators present. Fresh water was available *ad libitum*. Dogs were encouraged with vocal and food prompts to recline in a natural position with their right pelvic limb uppermost (Figure 1). Two investigators (one male and one female) were present during testing, however, application of the stimuli and recording of responses was always performed by the same male investigator (JRH).

#### 2.4.2 Sites of testing

Two sites were tested at each QST visit: a distal site located on the cranial aspect of the mid metatarsus between metatarsal bones III and IV (11) and a primary site, either 2 cm lateral to the patella (in dogs with stifle OA-pain) or 2 cm craniodorsally to the greater trochanter (in dogs with coxofemoral OA-pain). Control dogs were tested at appropriate primary sites to produce the same proportion of animals being tested at stifle or hip as those in the OA group. Preliminary data demonstrated no differences in QST measurements between left and right sides for animals affected by bilateral disease, therefore the decision was made to only test the right side in bilaterally affected animals to reduce the duration of the protocol which was thought to optimize the feasibility of testing. Sites were shaved with electric clippers prior to testing.

#### 2.4.3 Order of testing

The testing modalities were designated non-nociceptive (brush, tuning fork, air puff) and nociceptive [von Frey, Neuropen®, PROD, heat, cold, Canine Thermal Escape System (CTES)].

The order in which non-nociceptive and nociceptive tests were performed was randomized by pulling a scrabble tile out of a bag for each test site for each animal, although nociceptive tests were always performed after non-nociceptive tests. Non-nociceptive tests were performed at the distal site followed by the primary site, following which nociceptive tests were performed at the distal site followed by the primary site. This order of testing was employed in an attempt to reduce sensitisation of responses. A one-minute interval was allowed to elapse between non-nociceptive test replicates, and 5 min allowed to elapse between each nociceptive test replicate.

#### 2.4.4 Response evaluation

The outcome of fixed stimuli (air puff, application of brush, tuning fork, Neuropen®) was recorded from 0 to 3, graded according to a simple descriptive scale (SDS) (Table 1A), where 0 indicated no response and 3 indicated that the dog exhibited motor responses to avoid the stimulus and demonstrated awareness of the stimulus by orienting to the site of stimulation or vocalizing. The outcome of time dependent stimuli (heat, cold, vibration) was the latency to display a response (which could be from 1 to 3 on the SDS).

**Table 1A:** Simple descriptive scale (SDS) used to record degree of response to quantitative sensory testing stimuli.

SDS	Response
0	No response
1	Pay attention to stimulus (i.e., turn to look)
2	Withdraw limb in response to stimulus/move position to avoid stimulus
3	Withdraw limb/move position to avoid stimulus AND either pay attention to stimulus, vocalise, or get up from lying position.

#### 2.4.5 Test protocol

##### 2.4.5.1 Air puff

A standard bicycle pump was used to deliver an air puff perpendicular to the testing site 5 times at 10 s intervals. The outcome measure was the response to each puff.

##### 2.4.5.2 Brush

**Figure 2:**
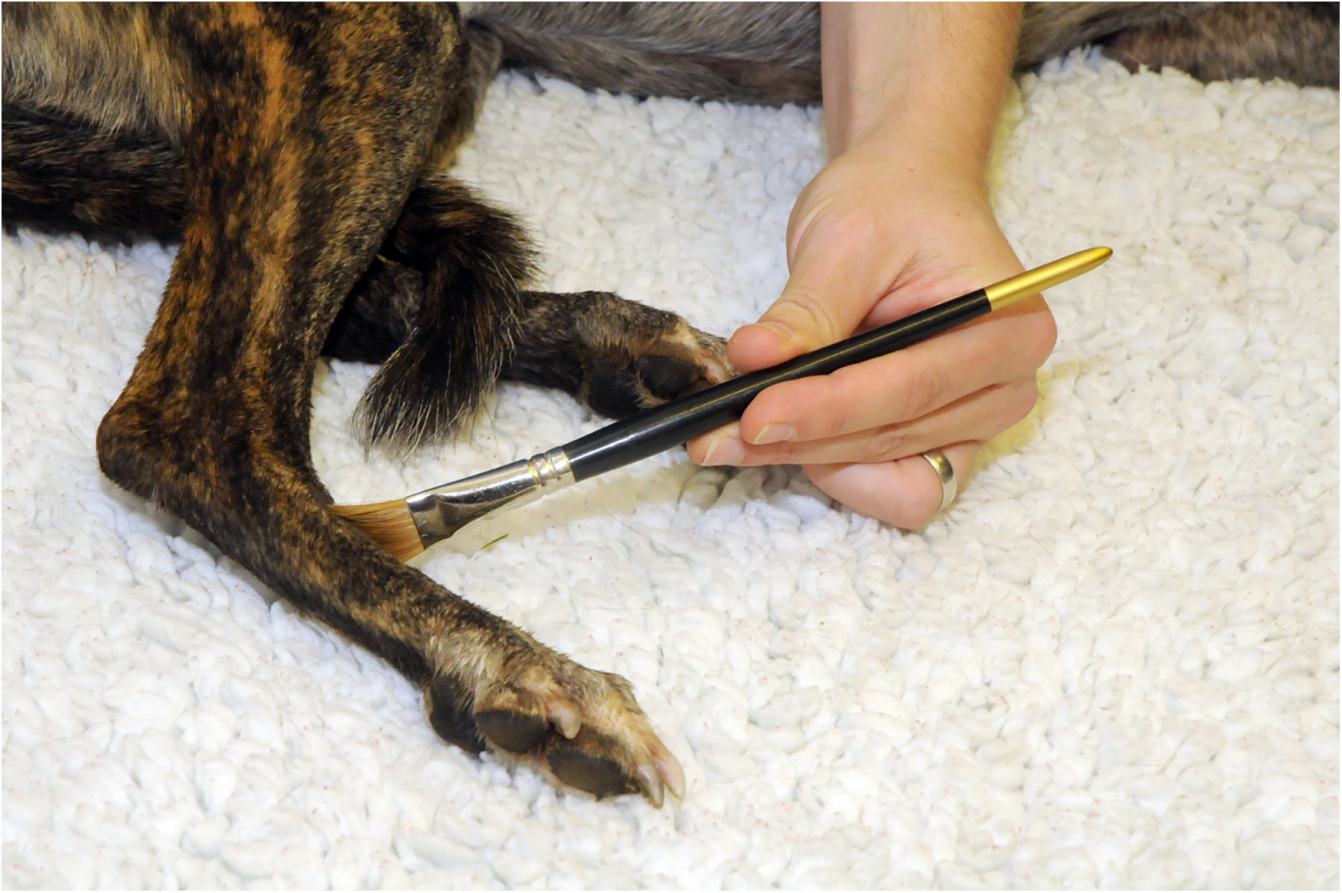
Brush stimulus applied to the dorsal metatarsal area of the limb using a paintbrush with a 1 cm wide flat head.

A paintbrush with a 1 cm wide flat head was used to lightly brush the skin of testing sites 5 times at 10 s intervals. The response to each application was recorded (Figure 2).

##### 2.4.5.3 Tuning Fork (Ragg Gardiner Brown Tuning Fork, Uniplex (UK) Ltd, Sheffield, UK)

**Figure 3:**
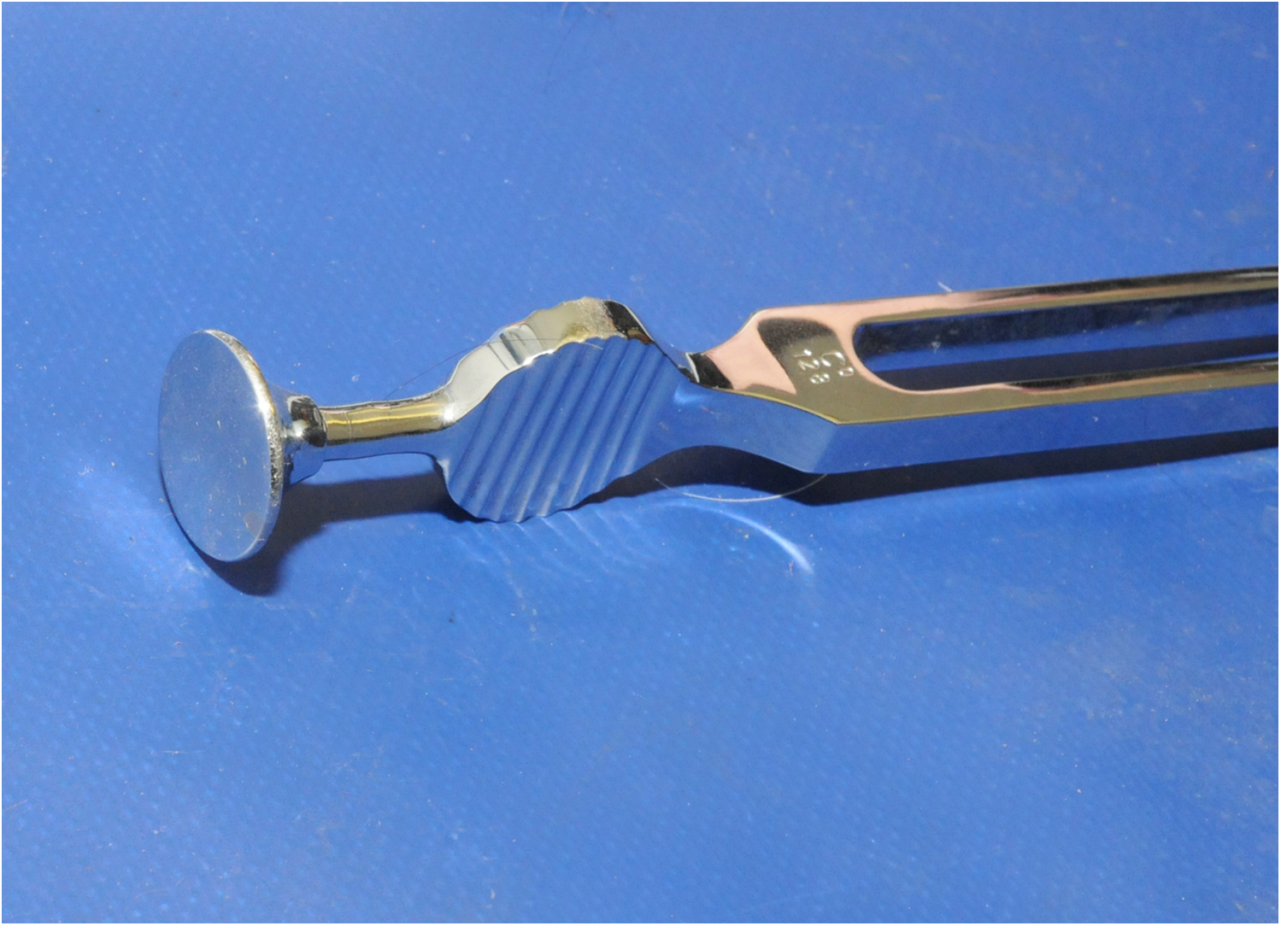
Tuning Fork used to test the response to vibration. The fork was struck on a rubber block and the flat base of the fork lightly held against the skin of the testing site.

A medical tuning fork (middle C, 128 Hz) was struck on a rubber block and the flat base of the fork lightly held against the skin of the testing site. The latency to respond to the stimulus, using a stopwatch accurate to 1/100th second (RS Components Ltd, Northants, UK), was recorded. If no response was observed at 15 s a latency of 15 s was recorded. The fork was applied a total of 5 times at 10 s intervals (Figure 3).

##### 2.4.5.4 Von Frey (Touch Test® Sensory Evaluators, North Coast Medical Inc., Ca, USA)

**Figure 4:**
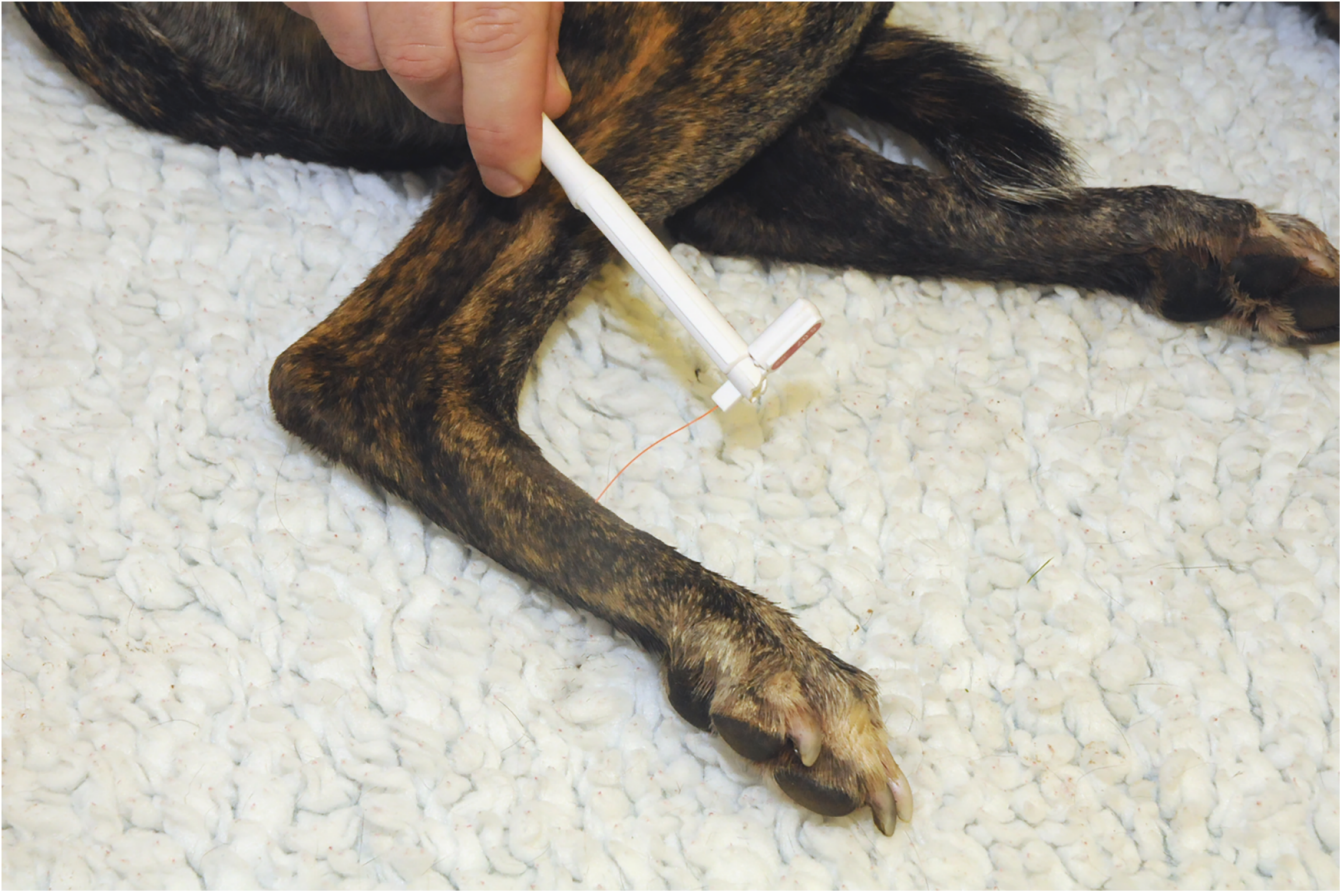
Von Frey filament applied to the dorsal metatarsal area of the limb.

A range of von Frey filament weights from 4 g to 300 g were applied to testing sites. Testing started with the lowest weight filament. If there was no response to stimulation the next highest weight filament was applied. If a response was elicited the next lowest weight filament was applied. This step method of detection was continued until 3 consistent responses were evoked at the same weight, which was recorded as the von Frey threshold. The threshold was determined once (Figure 4).

##### 2.4.5.5 Prod pressure algometer (ProdPlus, Topcat Metrology Ltd., UK)

**Figure 5:**
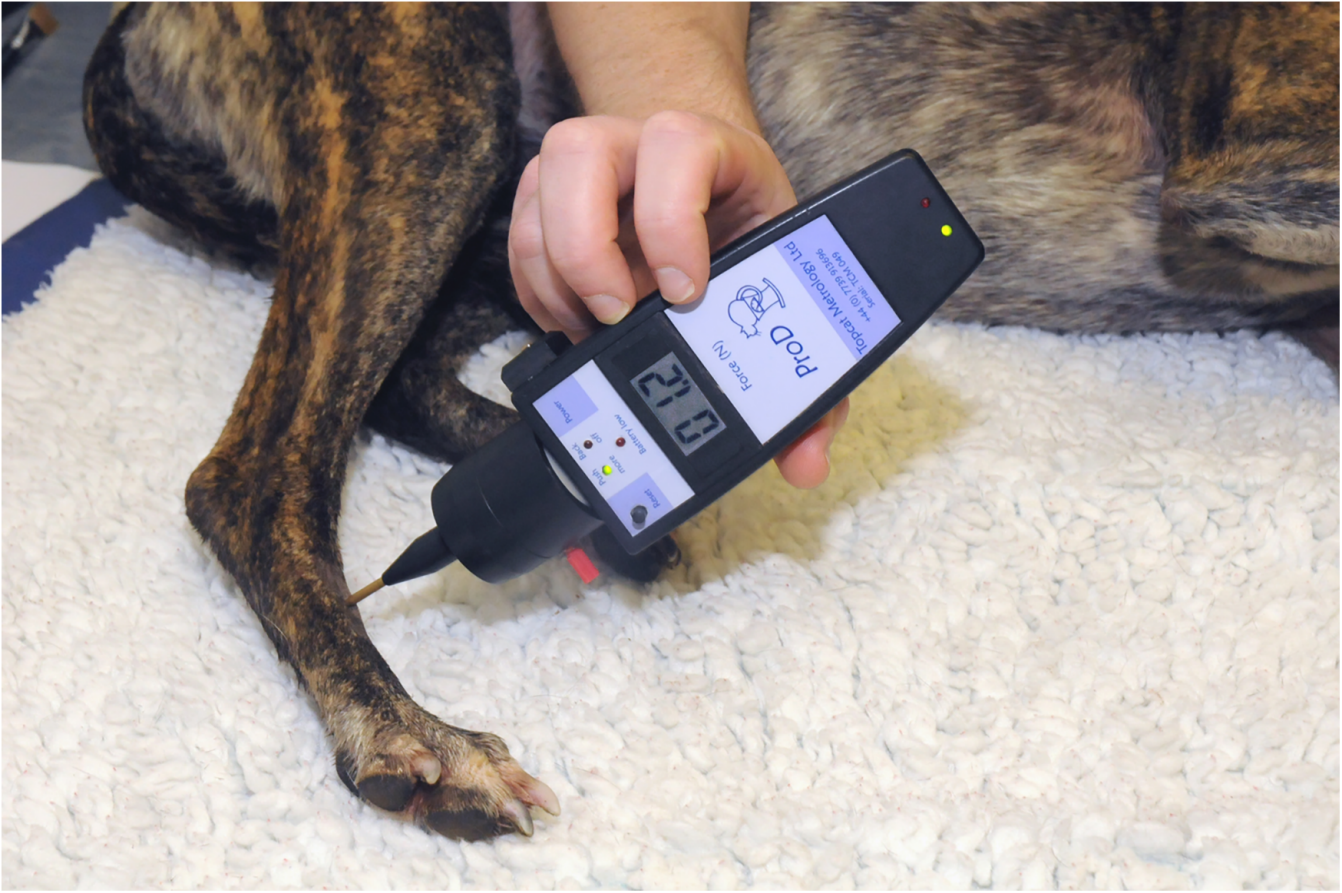
Pressure algometer applied to the dorsal metatarsal area of the limb.

The handheld PROD algometer was used with a 2 mm diameter flat tip to apply a ramped pressure stimulus at a rate of 2 Newtons (N) per second. The force at which a response was noted was recorded as the PROD pressure threshold. If no response was observed once the device had recorded an exerted force of 20N the test replicate was stopped and a result of 20N was recorded. The stimulus was repeated 3 times at 5-minute intervals (Figure 5).

##### 2.4.5.6 Neuropen® (Owen Mumford, Oxford, UK)

**Figure 6:**
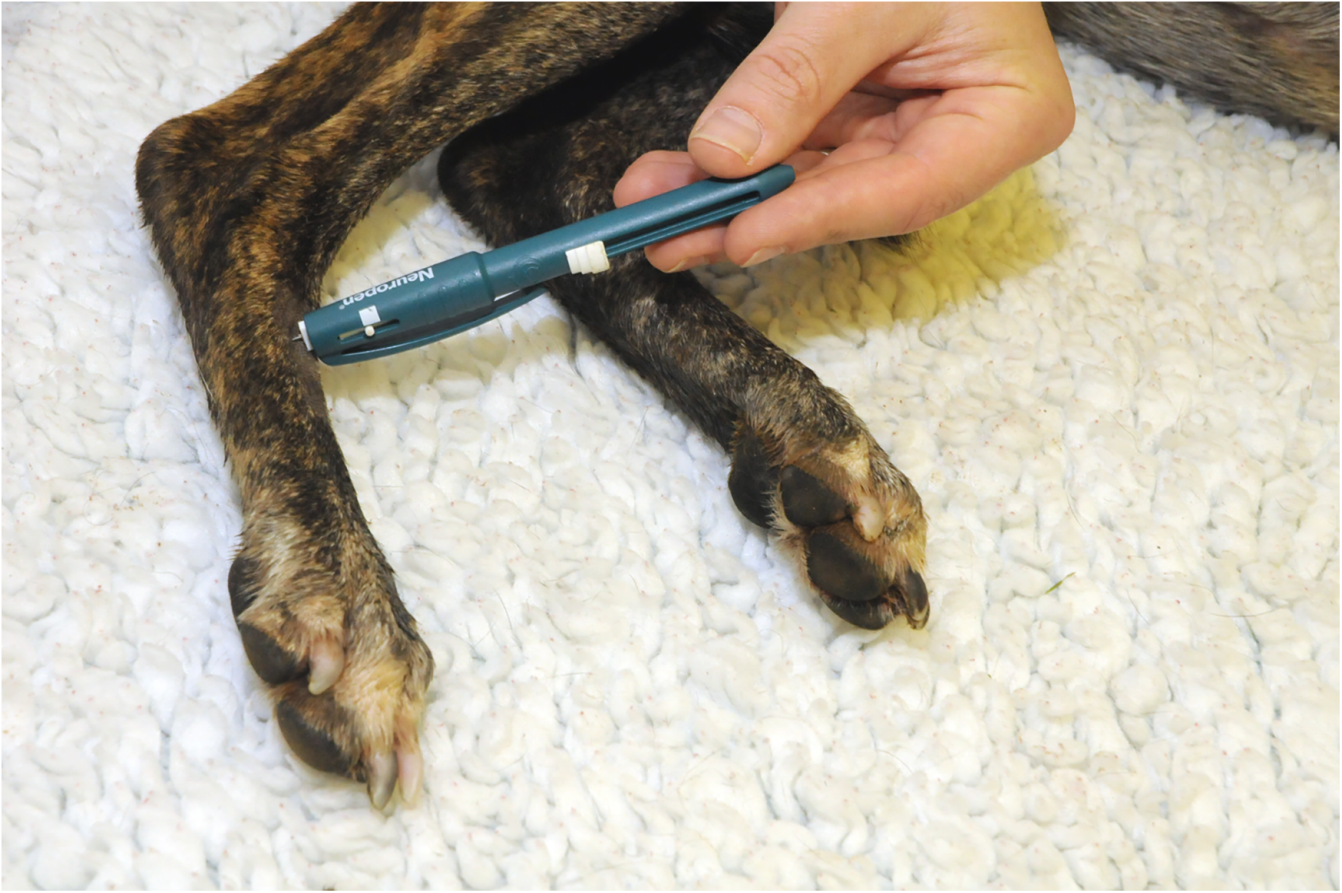
Neuropen® applied to the dorsal metatarsal area of the limb. The Neuropen® is a spring-loaded device which is designed to deliver a 40 g punctate stimulus, and was used to deliver a consistent stimulus for a period of 2 s.

The Neuropen®, which is a spring-loaded device designed to deliver a 40 g punctate stimulus, was used to deliver a consistent stimulus for a period of 2 s. The stimulus was applied 5 times at 1-minute intervals (Figure 6).

##### 2.4.5.7 Heat (hairy skin) (Physitemp controller NTE-2a, Physitemp Instruments Inc., NJ, USA)

The probe was heated to 49°C and applied to the test site, and latency to respond was recorded. A maximum stimulation time of 20 s was permitted. A total of seven applications [4 heated, 3 null (see below)] were applied at each test site at 1-minute intervals.

##### 2.4.5.8 Cold (hairy skin) (Physitemp controller NTE-2a, Physitemp Instruments Inc., Nj, USA)

**Figure 7:**
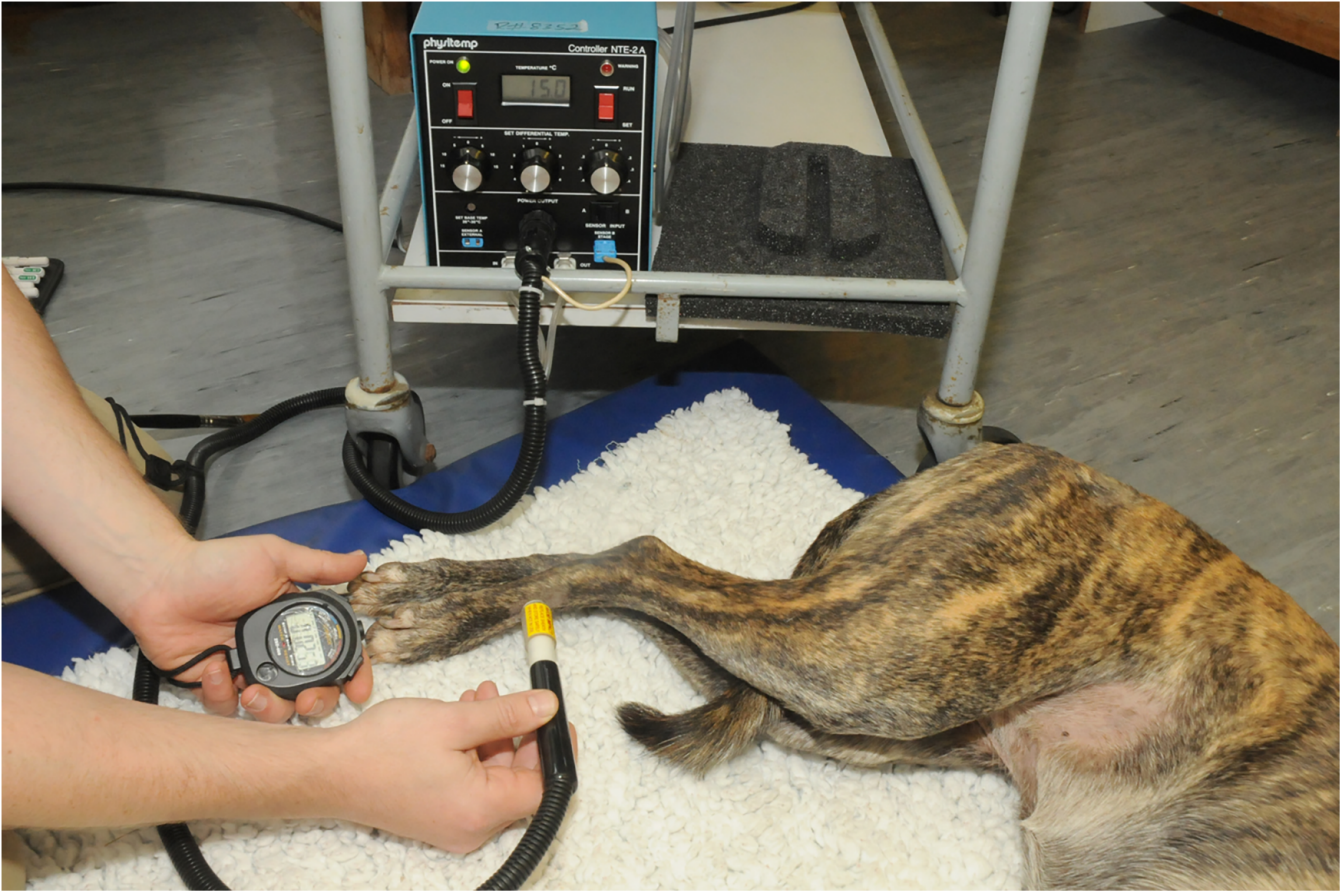
The physitemp device was used to deliver a hot and cold stimulus to the skin. The probe was set to 15°C for cold stimulus and 49°C for hot stimulus and applied to the test site, and latency to respond was recorded.

The probe was set to 15°C and applied to the test site, and latency to respond was recorded. If no response had occurred after 60 s, the probe temperature was reduced to 0°C. If no response had been elicited following 60 s at 0°C the test was terminated. A total of seven applications [4 cold, 3 null (see below)] were applied at each test site at 1-minute intervals (Figure 7).

##### 2.4.5.9 Canine thermal escape system (CTES) (hairless footpad) (Uc San Diego, University Anesthesia Research and Development Group, USA)

**Figure 8:**
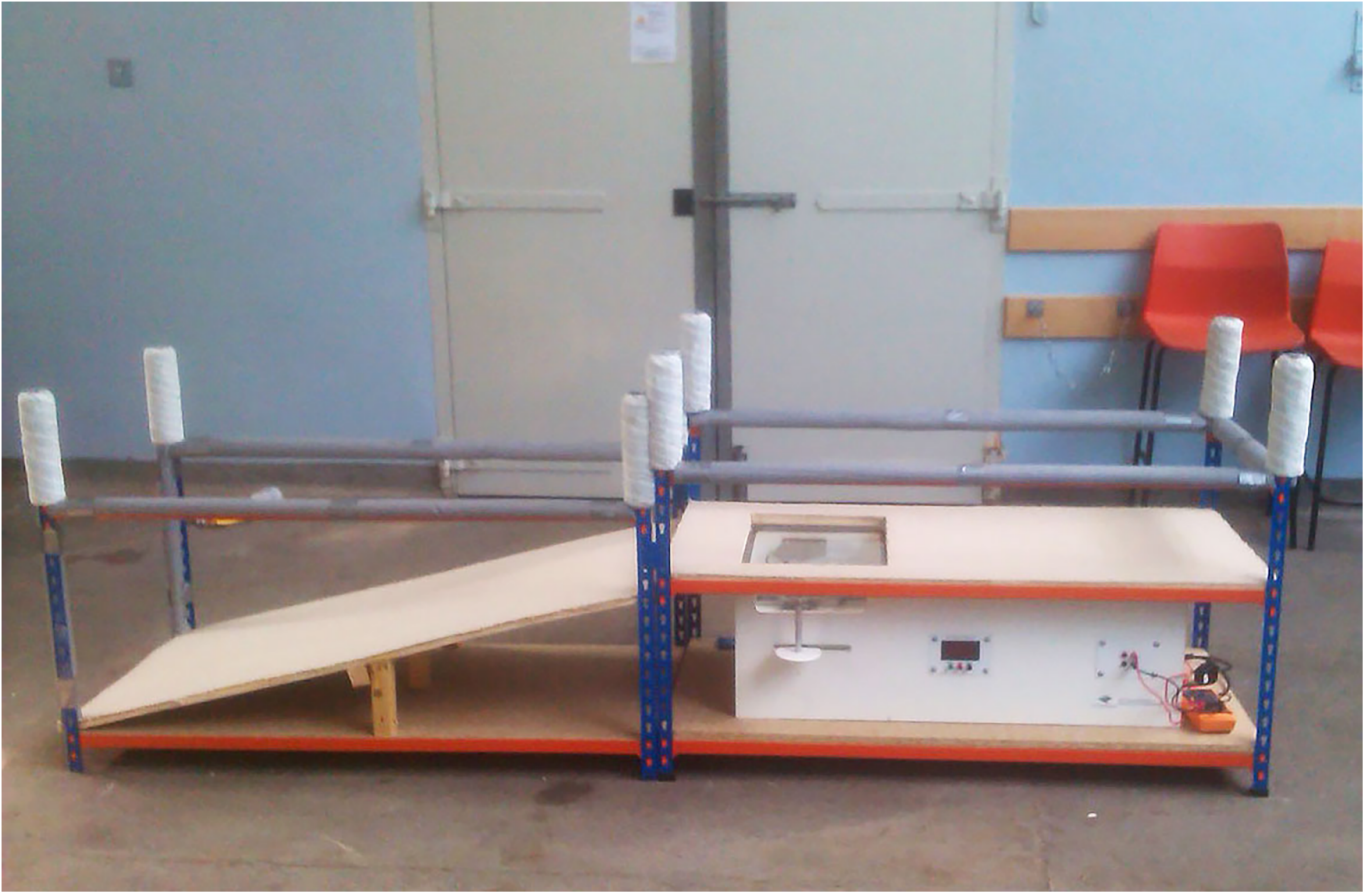
Canine thermal Escape system. Focused high-intensity lamps lie directly below the glass with positioning aids for placement of the dog's hind paws.

Dogs were encouraged to stand on the hotbox with both hindlimbs on the glass plate (12). The heating light source was positioned beneath the 3rd digital pad of the right hind foot and the latency to respond was recorded. A total of 5 applications (3 heat, 2 null) were applied at 1-minute intervals (Figure 8).

##### 2.4.5.10 Null stimuli

Within the heat, cold, and CTES tests, null stimuli were administered in order to gauge the animal's response to a non-noxious stimulus. For the null tests using the Physitemp the probe temperature was set to 34°C for the metatarsus site and 36°C for the hip site (based on preliminary data collected from skin temperature readings using a thermistor (RS206-3722 K-type thermocouple, RS components, Northants, UK). For the null tests using the CTES the location of the light source was moved so that heat was not directed within at least 15 cm of either foot. The order of noxious and null stimuli was randomized for each site.

#### 2.4.6 Feasibility

Feasibility was assessed for the final 45 dogs recruited to the study. Feasibility of performing the tests in their entirety was graded from 0 to 5 (12) (Table 1B), with a score of 1 indicating excellent feasibility with minimal restraint required and clear responses to stimuli, and 5 indicating that it was impossible to collect reliable data due to the dog's disposition or lack of confidence that the responses were elicited by the applied stimuli. A separate feasibility score using the same scale was assigned for the CTES. Scores of 0–2 were considered to represent acceptable feasibility for the testing protocol, whilst scores of 3 or greater represented unacceptable feasibility (12). There were 2 observers collecting the data and both agreed on the feasibility score.

**Table 1B:** Simple descriptive scale used to record feasibility of the quantitative sensory testing protocol.

Feasibility Score	Description
0 No problem	Minimum restraint needed; excellent cooperation; clear reaction to stimuli
1 Mild difficulty	Mild restraint needed; good cooperation; clear reaction to stimuli
2 Moderate difficulty	Moderate restraint needed; good cooperation >50% of the time; mild sensitivity of feet being touched; mild variation in reaction to stimuli
3 Significant difficulty	Significant restraint needed and resisted lateral recumbency; good cooperation <25% of the time; moderate sensitivity to feet being touched; moderate variation in reaction to stimuli
4 Extreme difficulty	Constant restraint required; not cooperative; unclear reaction to stimuli, not confident in data collected
5 Impossible	Could not collect data due to the dog's disposition and/or lack of confidence in the reactions seen being due to the stimulus

#### 2.4.7 Neurophysiological measures of spinal cord excitability

Significant differences were identified between painful OA and painful OANSAID groups, compared with control dogs, in characteristics of the A-fibre stimulus response, and C-fibre temporal summation data during our studies of the same cohort of dogs (19). Using these previous data, we derived summary values for use in the models. To derive individual summary measures of spinal cord excitability, the area under the early latency (A-fibre) stimulus response curve, and ratio of second to first late latency (C-fibre) responses during temporal summation stimulation (19) were calculated for each individual dog, and these measures were evaluated for their effects as predictor variables in the multi-level models describing the QST outcome measures (see statistical method, below).

### 2.5 Power calculation

A power calculation, based on preliminary data using von Frey mechanical threshold data, indicated a total of 68 dogs, evenly divided between OA and control groups, would be required for a power of 90%, at an alpha of 0.05 to detect a difference of 100 g between control and OA dogs. However, this calculation assumed uniformity within the OA group, whereas we suspected that the OA group would be heterogenous, based on data from human OA patients and laboratory animal models of OA. In humans, up to 70% of OA patients have at least one somatosensory abnormality (2). Based on this, we estimated that recruiting 100 OA dogs would give us an appropriate cohort of central sensitization (CS) negative dogs (i.e., approximately the same number as control dogs), and a cohort of CS positive dogs that may be as large as 70.

### 2.6 Statistical methods

Sex distribution data were assessed using Fisher's exact test. Comparisons of means or medians at single time points (e.g., body weight, owner completed metrology instrument scores) between the three groups were performed using one-way ANOVA or Kruskal–Wallis tests followed by Tukey (or Dunn's) *post-hoc* testing as applicable. The hierarchical structure of the QST testing data was accounted for within the statistical analysis by employing general linear modelling within a multilevel modelling framework using the MLwiN statistics package (25). Predictor variables were retained within the model based upon a Wald test at *α* ≤ 0.05. It was not necessary to transform the data for any of the outcome measures to meet the assumptions of the tests with regards to normality of errors and homoscedasticity. For each QST modality study a general linear modelling approach within a multilevel modelling framework was used to assess the statistical significance of the predictor variables. Where the outcome measure was on an integer scale (SDS, brush and air brush) a Poisson model was fitted. Potential predictor variables tested for significance within all of the models were body weight, age, OA category (control/OA/OANSAID), stimulus number (for tests including repeated stimuli), and neurophysiological measures (area under curve A-fibre stimulus response; ratio of 1st to 2nd C-fibre temporal summation). Site was entered into the models as a categorical variable, with the metatarsal site as the reference category, that is the category tied to the constant term within the models. Thus, the parameter estimates for the primary OA joint (stifle or hip) show their differences from the metatarsal site. Interactions between potential predictor variables were similarly examined. Data subject to parametric tests are presented as mean [95% confidence interval (CI)] and results subject to non-parametric testing are presented as median (25%–75% interquartile range).

## 3 Results

### 3.1 Demographics

Data were analysed from 30 control, 51 dogs with OA-associated pain (OA dogs), and 31 dogs with OA-associated pain and receiving NSAIDs (OANSAID dogs). There was no difference in sex distribution between groups (*p* = 0.59). Breed distribution is shown in Table 2 and appeared to be visually well matched between groups. Only 1 dog in each of the OA and OANSAID groups was diagnosed with primarily stifle OA, 1 control dog was assessed using the stifle joint as the primary reference joint.

**Table 2:** Demographic data for dogs in the control, OA and OANSAID groups.

Breed	Control (*n* = 30)	OA (*n* = 51)	OANSAID (*n* = 31)	*p*
Border collie	7	10	5	–
Labrador	5	8	13	–
Retriever	3	3	1	–
Lurcher	3	2	0	–
Spaniel	1	5	3	–
Other	11	23	9	–
Sex				
M	3	4	3	0.59
Mn	8	20	15	0.59
F	1	4	2	0.59
Fn	18	23	11	0.59
Body Weight (kg)	23.7 (95% CI 21.4–26.1)	27.2 (95% CI 24.2–30.1)	28.7 (95% CI 25.0–32.4)	0.10
Body condition score (1–9)	5 (4–6)	5 (5–6)	5 (4–6)	0.17
Age (years)	7.8 (95% CI 7.3–8.3)^a^	9.8 (95% CI 9.3–10.3)^b^	9.6 (95% CI 8.6–10.7)^b^	<0.001***

Dogs in the OA group had hip or stifle OA but were not receiving treatment with NSAIDs. Dogs in the OANSAID group had hip or stifle OA but were receiving treatment with NSAIDs. Different superscript letters indicate values that are statistically significantly different from each other.

*p ≤ 0.05; **p ≤ 0.01; ***p ≤ 0.001.

Body weight and body condition scores were not different between groups (Table 2). Dogs in the control group were significantly younger than dogs in both the OA and OANSAID groups (Table 2).

### 3.2 Veterinary assessment

Degree of lameness, mobility score, total OA score, and total joint pain score were all significantly higher in OA and OANSAID groups compared with controls (Table 3), however, there were no differences between OA and OANSAID groups with regard to these measures.

**Table 3:** Degree of lameness, mobility score, total osteoarthritis score, and total joint pain score in the control, OA and OANSAID groups.

Impairment and disease measures	Control	OA	OANSAID	*p*
Lameness (0–10)	0 (0–0)^a^	3 (1–3)^b^	3 (2–3)^b^	<0.001***
Mobility (0–3)	0 (0–0)^a^	1 (1–1)^b^	1 (1–1)^b^	<0.001***
Radiographic OA score (0–192)	0 (0–0)^a^	11 (7–13)^b^	13 (8–16)^b^	<0.001***
Joint pain score (0–48)	0 (0–0)^a^	4 (2–5)^b^	4 (2–6)^b^	<0.001***
CBPI pain (0–10)	0 (0–0.0625)^a^	1.5 (0.5–2.75)^b^	3.5 (2–4.8)^c^	<0.001***
CBPI function (0–10)	0 (0–0)^a^	2 (0.5–6)^b^	5.25 (2.25–7.94)^b^	<0.001***
HCPI (0–44)	2 (0–7.25)^a^	16 (11–22)^b^	19 (16–23)^b^	<0.001***
ACVS stiffness (0–16)	0 (0–0)^a^	5 (2–8)^b^	7.5 (5–10)^b^	<0.001***
ACVS function (0–16)	0 (0–0.25)^a^	4 (1–8)^b^	9 (5–12)^c^	<0.001***
ACVS gait (0–20)	0 (0–1.5)^a^	8 (4–12)^b^	10.5 (8–13)^b^	<0.001***
ACVS QoL (0–12)	0 (0–1)^a^	4 (2–5)^b^	5 (3–7)^b^	<0.001***
LOAD (0–52)	3 (0–7.25)^a^	15 (8–23)^b^	17.5 (12–23.5)^b^	<0.001***
Radiographic score (0–70)	3 (1–10)^a^	14 (8.25–24.75)^b^	20 (8–26)^b^	<0.001***

Different superscript letters indicate a statistically significant difference between groups.

*p ≤ 0.05; **p ≤ 0.01; ***p ≤ 0.001.

### 3.3 Owner completed metrology instruments

Questionnaire data were analysed by subsection if the questionnaire was constructed in a section format. Owner attributed scores for all of the questionnaire subsections were significantly higher (more dysfunction/pain) in OA and OANSAID animals compared with controls. Additionally, the CBPI pain and ACVS function subsections were significantly higher in OANSAID compared to OA animals (Table 3).

### 3.4 Radiographic OA scores

Radiographic OA severity was significantly higher in both OA and OANSAID animals compared with controls but was not significantly different between OA and OANSAID animals (Table 3).

### 3.5 Feasibility

Feasibility was assessed for 17 control, 15 OA, and 13 OANSAID dogs. Feasibility was not different between groups (*p* = 0.84). At QST session 1, 56% of tests were considered feasible (scores 0–2), whilst at QST session 2, 73% of tests were feasible; statistically these proportions were not different (*p* = 0.11).

### 3.6 QST results

#### 3.6.1 Non-nociceptive tests

Dogs in OA and OANSAID categories demonstrated significantly reduced SDS responses to the air puff stimulus, compared with controls (*p* = 0.04).

Stimulation at the hip in all three of the tests, and additionally at the stifle in the air puff and tuning fork tests, was associated with a decreased response (either longer latency to respond or reduced SDS response) compared with stimulation at the metatarsal site.

Increasing repetition of the brush stimulus (from 1 to 5) was associated with a decreasing SDS response [−0.02 (95%CI −0.036 to −0.012), *p* < 0.001].

As body weight increased, the response at the metatarsus site to the tuning fork stimulation decreased (latency to respond increased) [0.12 (95% CI 0.05–0.18) seconds, *p* < 0.001], however, at the hip site this effect was countered by a negative interaction between body weight and the site (response latency remained largely constant at the hip, close to the maximum latency of 15 s, irrespective of dog body weight) (Table 4).

**Table 4:** Parameter estimates, standard error (SE), and *p* values for the general linear models fitted to the outcome measures (specified in the table) for the non-nociceptive tests.

Row variable	Airpuff	S.E.	*p*-value	Brush	S.E.	*p*-value	Tuning fork	S.E.	*p*-value
Response	Air puff response 0–3			Brush response 0–3			Response latency (seconds)		
Constant	0.72	0.08	<0.001***	0.28	0.03	<0.001***	7.13	0.94	<0.001***
Stifle	−0.51	0.26	0.048*	−0.08	0.14	0.59	7.72	3.94	0.05*
Hip	−0.30	0.05	<0.001***	−0.12	0.04	<0.001***	5.89	0.99	<0.001***
OA	−0.21	0.10	0.040*	–	–	–	–	–	–
OANSAID	−0.28	0.11	0.013*	–	–	–	–	–	–
Stimulus number	–	–	–	−0.02	0.006	<0.001***	–	–	–
Weight	–	–	–	–	–	–	0.12	0.03	<0.001***
Stifle.weight interaction	–	–	–	–	–	–	−0.16	0.13	0.22
Hip.weight interaction	–	–	–	–	–	–	−0.13	0.04	<0.001***

For the categorical variables the reference category was control, and metatarsus was the reference site. – indicates variables were not significant and were dropped from the model.

* *p* ≤ 0.05; **p ≤ 0.01; *** *p* ≤ 0.001.

#### 3.6.2 Nociceptive tests

##### 3.6.2.1 Mechanical

**Figure 9:**
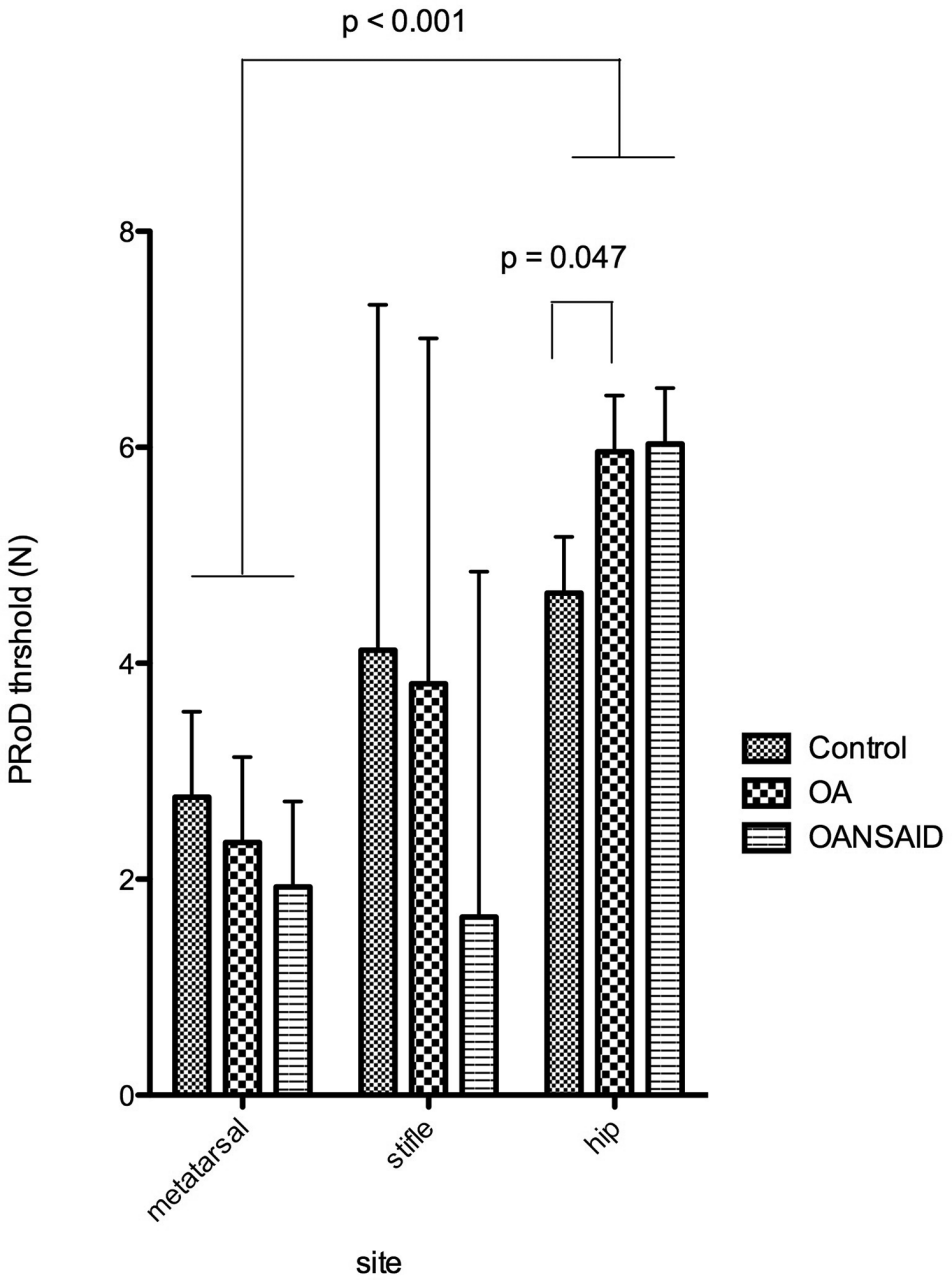
Mechanical nociceptive threshold measured using the pRoD algometer at the three test sites - dorsal metatarsal area, the stifle and the hip. Dogs were divided into three groups depending on whether they were a control animal, had osteoarthritis but were not receiving NSAIDs (OA) or had OA and were receiving NSAIDs (OANSAIDs group). Data are shown as mean ± SEM.

The PROD threshold at the hip in OA dogs was significantly higher than that of control dogs (+1.3 95% CI 0.02–2.6 N; *p* = 0.047); whilst the hip threshold in OANSAID dogs demonstrated a similar increase in magnitude, although this was not quite significant compared with controls (+1.4 95% CI −0.06–2.8 N; *p* = 0.06). Osteoarthritis status had no effect on thresholds and responses at the metatarsus for any of the mechanical stimuli (Figure 9).

For all of the dogs, irrespective of disease category, von Frey (+148 95% CI 121–177 g; *p* < 0.001) and PROD (+1.9 95% CI 0.9–2.9 N; *p* < 0.001) thresholds at the hip were significantly elevated above the reference metatarsus results (135 95% CI 106–165 g; 2.8 95% CI 1.2–4.3 N respectively). Response to the Neuropen® was significantly decreased at the stifle and hip compared with the metatarsus. In association with increasing body weight, PROD nociceptive threshold at all sites increased (+0.06 95% CI 0.01–0.11 N; *p* = 0.015), and response to Neuropen® stimulation at the metatarsus site decreased (Table 5).

**Table 5:** Parameter estimates, standard error (SE), and *p* values for the general linear models fitted to the outcome measures (specified in the table) for the nociceptive tests.

Response	Von frey threshold (grams)			PRoD threshold (newtons)			Neuropen response (0–3)			Response latency (seconds)			Response latency (seconds)			Response latency (seconds)		
	von Frey	S.E.	*p*-value	Prod	S.E.	*p*-value	Neuro pen	S.E.	*p*-value	Heat	S.E.	*p*-value	Cold	S.E.	*p*-value	CTES	S.E.	*p*-value
Constant	135.56	15.08	<0.001***	2.76	0.79	<0.001***	1.27	0.15	<0.001***	13.17	0.98	<0.001***	31.43	7.22	<0.001***	16.62	1.77	<0.001***
Stifle	105.99	72.64	0.14	1.36	3.20	0.67	−1.24	0.63	0.05*	−0.55	1.91	0.78	12.74	12.25	0.30	–	–	–
Hip	148.82	14.37	<0.001***	1.89	0.52	<0.001***	−0.81	0.16	<0.001***	0.43	0.47	0.37	7.16	2.74	0.009**	–	–	–
OA	–	–	–	−0.42	0.68	0.53	–	–	–	–	–	–	–	–	–	−0.68	0.98	0.49
OANSAID	–	–	–	−0.83	0.76	0.27	–	–	–	–	–	–	–	–	–	−0.86	1.09	0.43
Stimulus number	–	–	–	–	–	–	–	–	–	–	–	–	–	–	–	−0.26	0.12	0.04*
Weight	–	–	–	0.06	0.03	0.015*	−0.02	0.005	<0.001***	0.09	0.03	0.005**	0.65	0.25	0.009**	0.07	0.07	0.32
Stifle.weight interaction	–	–	–	–	–	–	0.021	0.021	0.31	–	–	–	–	–	–	–	–	–
Hip.weight interaction	–	–	–	–	–	–	0.014	0.006	0.013**	–	–	–	–	–	–	–	–	–
OA. Stifle interaction	–	–	–	−0.31	3.80	0.94	–	–	–	–	–	–	–	–	–	–	–	–
OANSAID. Stifle interaction	–	–	–	−2.47	3.91	0.53	–	–	–	–	–	–	–	–	–	–	–	–
OA.Hip interaction	–	–	–	1.31	0.66	0.047*	–	–	–	–	–	–	–	–	–	–	–	–
OANSAID. Hip interaction	–	–	–	1.38	0.74	0.06	–	–	–	–	–	–	–	–	–	–	–	–
Nociceptive stimulus (as opposed to null)	–	–	–	–	–	–	–	–	–	−4.00	0.38	<0.001***	−10.58	1.59	<0.001***	−6.75	1.48	<0.001***
Stifle. nociceptive stimulus interaction	–	–	–	–	–	–	–	–	–	1.76	1.73	0.31	–	–	–	–	–	–
Hip. nociceptive stimulus interaction	–	–	–	–	–	–	–	–	–	1.92	0.52	<0.001**	–	–	–	–	–	–
OA. nociceptive stimulus interaction	–	–	–	–	–	–	–	–	–	–	–	–	–	–	–	−1.76	0.84	0.04*
OANSAID. nociceptive stimulus interaction	–	–	–	–	–	–	–	–	–	–	–	–	–	–	–	−1.29	0.93	0.17

For the categorical variables the reference category was control, and metatarsus was the reference site. – indicates variables were not significant and were dropped from the model.

* *p* ≤ 0.05; **p ≤ 0.01; *** *p* ≤ 0.001. CTES is Canine Thermal Escape System.

##### 3.6.2.2 Thermal

Application of a nociceptive stimulus, compared with a null stimulus, decreased the latency for thermal (−4.0 95% CI −4.7 to −3.2 s), cold (−10.6 95% CI−13.7 to−7.5 s), and CTES (−6.8 95% CI −9.6 to −3.9 s), all *p* < 0.001.

**Figure 10:**
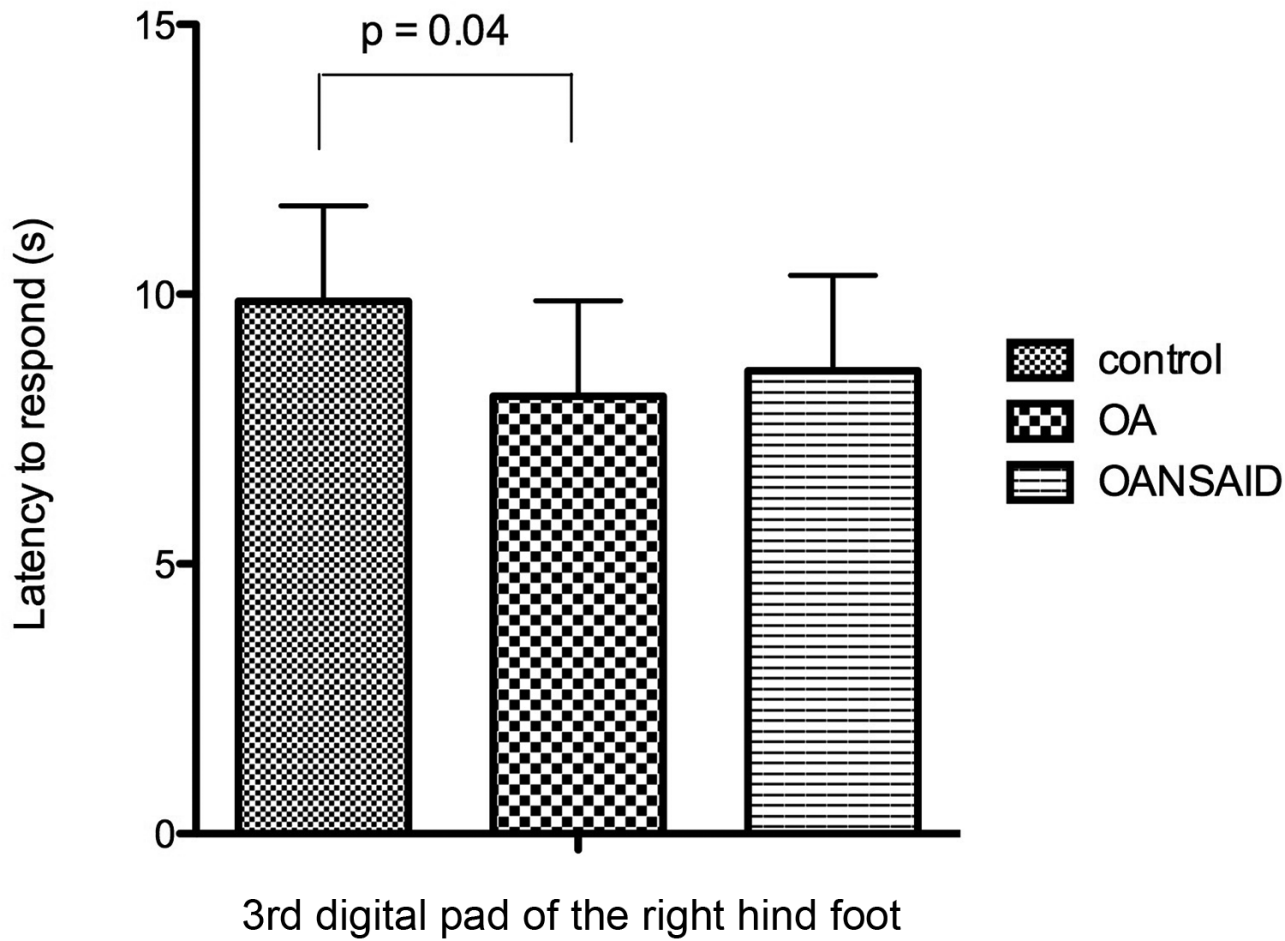
Latency of dogs to respond with a foot lift when testing with the canine thermal escape system (CTES). Dogs with OA that were not receiving NSAIDs (OA group) had a significantly shorter time to respond to the heat stimulus than control animals. Differences between animals with OA that were receiving NSAIDs (OANSAIDs group) and control animals were not statistically significant. Data are shown as mean ± SEM.

Dogs in the OA group demonstrated decreased latency to respond to nociceptive stimuli during the CTES test compared with control dogs (OA.nociceptive stimulus interaction; −1.8 95% CI −3.4 to −0.12 s; *p* = 0.04) (Figure 10). Although dogs in OANSAID demonstrated similar magnitude decreases in latency to respond, these changes were not significantly different to control (−1.3 95%CI −3.1- 0.53 s; *p* = 0.17). OA status did not predict response to heat or cold stimulation evaluated using the Physitemp apparatus.

The latency to respond to hot and cold Physitemp stimulation at the hip was increased, compared to the metatarsus. Latency to respond during heat and cold tests increased in association with increasing body weight (+ 0.09 95%CI 0.03–0.16 s; *p* = 0.005 and +0.6 95%CI 0.16–1.1 s; *p* = 0.009 respectively), although no effect of body weight was evident for the CTES test (*p* = 0.32). Increasing stimulus number was associated with a shorter latency to respond during the CTES test (−0.3 95% CI −0.5 to −0.02 s; *p* = 0.04).

#### 3.6.3 Association of QST outcome measures with neurophysiological measures

The summary individual neurophysiological measures were not found to have significant effects as predictor variables on outcome measures in any of the models.

## 4 Discussion

In contrast to our hypothesis, OA category was not a major determinant of QST outcome measures for the majority of modalities evaluated and, even when significant differences were detected between dogs with OA and the control group, the separation between the groups was minimal so that at the level of the individual animal the QST modalities were poor diagnostic tests. This was surprising, given that other research groups have identified somatosensory changes indicative of CS in dogs suffering from OA through the application of similar QST methods (12, 14, 17), and that we were able to identify group level alterations in neurophysiological responses, consistent with CS, in the OA and OANSAID cohort studied here (19). One potential factor could be that there is severe data heterogeneity among QST studies in dogs (26). In the few modalities in which OA category was determined to be a significant predictor variable, results were mixed compared with previously reported data. Our results were suggestive of an increased mechanical nociceptive threshold (primary hypoalgesia) at the hip in OA affected dogs compared with controls (in contrast to previous work), however we found a decreased latency to respond to thermal stimulation of the plantar digital pad (secondary hyperalgesia) (consistent with some previous work) (14). One of the novel tests (air puff) demonstrated decreased responses in both OA and OANSAID dogs, and may reflect tactile hypoalgesia. Knazovicky et al. (2016) (14) demonstrated decreased mechanical threshold to both deep pressure and von Frey stimuli at both primary and secondary sites in OA affected dogs, findings which correlate with reported increased mechanical sensitivity in human patients suffering from OA (27, 28).

Thermal hypoesthesia, as evaluated by the CTES, has been reported in dogs suffering from OA, compared with control dogs (12), whilst our results are suggestive of increased heat pain sensitivity using this apparatus. Williams et al. postulated that their results may be indicative of dogs with painful joints not wanting to shift weight and lift the hindlimb in response to the stimulus. One study in humans reported that heat pain thresholds at both primary and secondary sites were not different in OA compared with controls (2), whilst other investigators (29) have reported decreased heat pain thresholds associated with OA (i.e., thermal hyperalgesia). In humans it has been suggested that subgroups of QST phenotype, identifiable by cluster analysis (30, 31), exist in both OA affected (32) and healthy (33) populations, therefore comparing outcome measures between OA and control groups might not be expected to reveal differences between the populations, as inter-individual variability may be relatively more important than disease status. The importance of individual (dog) above other factors as a source of variability in determining mechanical nociceptive threshold in healthy dogs has been reported (34) and may reflect the presence of individual QST phenotypes in dogs.

Knazovicky et al. (2016) (14) determined that feasibility was a significant source of variability of the thresholds recorded for von Frey and heat stimulation at the primary site, therefore it is possible that the generally lower feasibility of testing which we recorded has impacted the measured thresholds, and confounded differences between groups. The proportion of QST assessment attempts in client owned dogs which have been completed with acceptable feasibility has previously been reported in the order of 80% (12, 35), however, only 56% of the first QST sessions in the present study were considered to have acceptable feasibility, although this increased to 73% for second QST sessions. Harris et al. (2015) (34) reported a number of factors which influenced the response rate to a mechanical stimulus, finding that a tip diameter of 2 mm (as used in the present study), and performance of the test with the dog in a sitting position improved the ability to record a response, however, application at both metatarsus and hip sites was not evaluated in that study, and it would have been difficult to achieve application of stimuli to the dorsal surface of the metatarsus whilst the hock was flexed, in a sitting position. Briley et al. (2014) (35) attained a proportion of 83% feasible mechanical nociceptive tests with dogs lying in lateral recumbency, which suggests that aspects of testing in our protocol (which was designed to mimic conditions in a quiet room within a shared building as would be a common scenario in clinical veterinary practice) adversely affect feasibility. The occurrence of stimuli within the shared building, even if not impinging on the testing room, appeared to result in distraction of the dogs from the testing. A large proportion would stand up and approach the door of the testing room in response to hearing entrants to the building. Stimuli related to housekeeping, occurring within the building but outside of the testing room, would cause a noticeable alteration in attention of some of the dogs. Rarely did people walking past the window seem to capture the attention of dogs. It is likely that minimising extraneous stimuli is necessary to achieve good feasibility during testing protocols that are dependent on behavioural outcomes.

Feasibility was only assessed in a minority of dogs within the study, however, the distribution between groups, and demographics of the assessed dogs, were representative of the population as a whole. It is likely that there was insufficient statistical power provided by this number of samples to evaluate an improvement in feasibility from the first to second QST session. However, a change from 56% to 73% acceptable feasibility would suggest a clinically relevant difference and may suggest that familiarity with the test environment is required over a greater period than that offered by the initial 15-min acclimatisation.

Before QST can be recommended as a clinical or translational research tool a thorough quantification of factors affecting the feasibility is necessary. Video analysis of testing protocols, coupled with time locked information on external stimuli, may provide a beneficial overview of factors which need to be studied more closely.

Both the von Frey filaments and the Electronic von Frey Anesthesiometer may be used for this purpose. Study in cats showed that performing QST with both the Electronic Von Frey and Von Frey Filaments is feasible, and that the sensory thresholds measured at the lip and at the stifle with these two algometers are comparable, indicating fair interdevice agreement (36). Due to its practicality, broader numerical range, and reduced need for repeated applications, the Electronic von Frey may be considered a superior option for QST compared to Von Frey Filaments.

Self-report of sensations associated with QST in verbal humans enables the relatively straightforward detection of subtle changes, for example with regard to detection threshold, and permits the evaluation of differing degrees of pain perception, for example pain threshold and pain tolerance. In contrast, behavioural measures in animals must be employed as surrogate markers for awareness of stimulation, and are unable to definitively differentiate between nociceptive responses/reflexes, and the conscious experience of the feeling of pain. Moreover, responses in awake animals may reliably indicate nociceptive (or pain) thresholds, or may be over-ridden by tolerance of the nociceptive stimulus (14) that could vary between dogs. The companion role of dogs and associated desirable trait of calmness/compliance (37) (which may manifest as a willingness to appease humans), may also have ramifications with regard to their response to a nociceptive stimulus, particularly one associated with a human investigator as in the case of a hand-held device.

Decreasing response to the brush with increasing stimulus number may represent learned tolerance to a non-nociceptive stimulus, whilst decreased latency to the CTES with increasing stimulus number may represent a learned response to a nociceptive stimulus. Decreases in threshold assumed to be learned/anticipatory behaviour in association with repeated mechanical nociceptive stimulation have previously been documented (38), therefore it may be preferable to utilise tests employing single stimuli to quantify responses, without the confounding effects of learning.

Body weight has previously been reported to be a significant factor in the determination of mechanical nociceptive stimuli (34, 35), and our results suggest that, additionally, vibration and thermal thresholds also vary with body weight. This finding corroborates the evidence produced in our electrophysiological studies (19), and suggests that publication of normal ranges for QST measures will require a correction factor for body weight.

In summary, the techniques as described in this study suggest that, in a clinical setting, variability in the results between dogs may be substantial enough to obscure any changes associated with CS. Consequently, the methods cannot currently be recommended for evaluating CS in dogs. Other methodology and objective measures such as the nociceptive withdrawal reflex, should be explored. Additionally, the application of stimuli that appear remote from the investigator, as seen with CTES, may produce more meaningful data (in terms of indicated altered central processing of noxious stimuli) compared to those delivered via hand-held devices.

## Data Availability

The raw data supporting the conclusions of this article will be made available by the authors, without undue reservation.
